# Mapping Central Projection of Oxytocin Neurons in Unmated Mice Using Cre and Alkaline Phosphatase Reporter

**DOI:** 10.3389/fnana.2020.559402

**Published:** 2020-10-19

**Authors:** Po-Yu Liao, Yan-Min Chiu, Jo-Hsien Yu, Shih-Kuo Chen

**Affiliations:** ^1^Department of Life Science, National Taiwan University, Taipei, Taiwan; ^2^Genome and Systems Biology, National Taiwan University and Academia Sinica, Taipei, Taiwan; ^3^Center for Biotechnology, National Taiwan University, Taipei, Taiwan

**Keywords:** oxytocin, innervation, naïve mouse, Cre-loxP, circuit

## Abstract

Oxytocin, a neuropeptide and peptide hormone, is produced by neurons in the hypothalamus and released by the posterior pituitary to control breastfeeding and labor. Recent studies have revealed that oxytocin in the central nervous system is also involved in modulating social interaction. To understand the potential role and innervation pattern of oxytocin neurons before sexual interaction, here we used transgenic mice which have the Cre recombinase under the control of an endogenous oxytocin promoter and Cre-dependent human placental alkaline phosphatase (AP) reporter to label the oxytocin neurons in the naive mouse brain. Since AP is located on the membrane of oxytocin neurons, AP histochemistry staining enabled us to observe the fine axonal terminals and the innervation pattern of oxytocin neurons in the thick serial coronal brain slices. Here we show that the number of AP-labeled cells varies with staining reaction time and ranges from 30% of the oxytocin immune-positive cell count to slightly higher than the oxytocin immune-positive cell count. Using AP staining with extended reaction time, which may not label all oxytocin neurons, we confirmed many innervation targets of oxytocin neurons from the anterior olfactory nucleus, some cortex regions, the limbic system, the hypothalamus, and the hindbrain, while the cell bodies were exclusively located in the hypothalamus and the bed nucleus of the stria terminalis. Finally, we observe some individual variance at the olfactory area, isocortex, striatum, paraventricular nucleus of thalamus, locus coeruleus, and Barrington’s nucleus.

## Introduction

In recent years, increasing numbers of studies have shown that oxytocin, the polypeptide neurotransmitter/hormone, plays versatile roles in regulating both physiological and social functions (Donaldson and Young, 2008; Stoop, 2012; Wu et al., 2012). It is well known that oxytocin is mainly released from neurons at the paraventricular nucleus (PVN) and the supraoptic nucleus (SON) in the hypothalamus (Lee et al., 2009; Rhodes et al., 1981). These oxytocin neurons can be divided into magnocellular cells and parvocellular cells, depending on the morphology and the electrophysiological characteristics (4–6), or genetically divided into four different types (Romanov et al., 2017). Traditionally, it is known that magnocellular cells mainly project to the posterior pituitary glands and release oxytocin in the bloodstream to influence peripheral functions as a neuronal hormone, while parvocellular cells project to the midbrain and the spinal cord to control autonomic functions. In addition, many studies have shown that oxytocin neurons also have central projections and may influence the central nervous system through dendritic and somatic release (Freund-Mercier and Stoeckel, 1995; Ludwig, 1998; Knobloch et al., 2012). However, the detailed function of oxytocin in the central nervous system has still not been fully investigated.

According to the expression patterns of oxytocin receptors and the innervation patterns of oxytocin neurons, it has been suggested that oxytocin neurons could have a complex central projection to regulate animal behavior or physiological responses (Donaldson and Young, 2008; Nasanbuyan et al., 2018). Traditional immunohistochemistry staining methods have been used to study oxytocin neuron innervation in rodents. Recently, detailed studies using virus vectors injected to the SON and PVN in rats (Eliava et al., 2016) or mice (Xiao et al., 2017) also showed many central projection targets. It has been shown that OXT fibers originated from the PVN and SON in the hypothalamus together innervate rostrally up to the anterior olfactory nucleus (AON) and caudally down to the medulla and the spinal cord. Briefly, in the hypothalamus, in addition to the SON and PVN, many nuclei in the preoptic area (POA) have OXT neuron cell bodies and high density of fibers (Rhodes et al., 1981; Castel and Morris, 1988). In the thalamus, OXT fibers could be observed in the paraventricular nucleus of the thalamus (PV) (Knobloch et al., 2012; Yao et al., 2017; Nasanbuyan et al., 2018). In cortical areas, a low density of projection and possible terminals were observed in the orbital cortex, prelimbic cortex (PrL), tenia tecta (TT), and the cingulate cortex (Cg) (Sofroniew, 1983a,b; Knobloch et al., 2012; Nasanbuyan et al., 2018). In the limbic system, fibers can be observed in the bed nucleus of stria terminals (BST), the amygdala, the septal nucleus, the hippocampus, the nucleus accumbens (AcB), and the caudate putamen (Cpu) (Sofroniew, 1983a; Knobloch et al., 2012; Nasanbuyan et al., 2018). In the midbrain and the hindbrain, OXT fibers innervated to several regions such as the periaqueductal gray (PAG), ventral tegmental area (VTA), substantia nigra (SN), locus ceruleus (LC), and raphe nucleus (Sofroniew, 1980; Sofroniew, 1983a; Nasanbuyan et al., 2018). In the medulla and the spinal cord, oxytocin-immunoreactive fibers and terminals could also be detected (Sofroniew, 1980; Sofroniew, 1983a). In addition to the brain regions listed above, genetic labeling using virus vectors confirms the aforementioned findings, and OXT innervation can be observed from different origins in several downstream projection sites (Sofroniew, 1980, 1983a,b; Castel and Morris, 1988; Knobloch et al., 2012; Yao et al., 2017; Nasanbuyan et al., 2018). Although both methods provide similar innervation patterns, with some variation of branching intensity in the target brain area, there are some limitations to these studies. The sensitivity of immunohistochemistry is relatively low compared to a Cre-dependent genetic reporter, thus a fine detailed pattern is hard to observe. Although the direct injection of a virus-carrying reporter to the SON or PVN could provide a stronger labeling signal, it is hard to label all oxytocin expression neurons using a virus vector, and it may lack additional targets from few oxytocin neurons outside of SON and PVN.

In order to study the fine innervation patterns of oxytocin neurons in mice, we labeled the oxytocin neurons using the Cre-loxP system by crossing transgenic mouse lines with Cre oxytocin Cre knockin mice (Wu et al., 2012) and floxed alkaline phosphatase (AP) reporter (Z/AP) (Lobe et al., 1999). Combining the specificity of the genetic oxytocin Cre line and the strong staining sensitivity provided by AP reporter, we could construct the innervation pattern of oxytocin neurons from the whole mouse brain.

## Materials and Methods

### Animal Models

Transgenic mouse line Z/AP (Z/AP) (Lobe et al., 1999; Chen et al., 2011) maintained in C57BL/6 background (National Laboratory Animal Center, TW) was crossed with B6;129S-*Oxt*^*TM* 1^.^1(Cre)Dolsn^/J (Oxt^Cre^) (Wu et al., 2012) (JAX stock #024234) to obtain Oxt^*Cre/+*^; Z/AP mice for the experiments. The *Oxt*^Cre^ was genotyped by polymerase chain reaction analysis of genomic DNA extracted from mouse tails using the primers 5′-ACACCGGCCTTATTCCAAG-3′, 5′- TTTGCAGCTCAGAACACTGAC-3′, and 5′-AGCCTGCTGGACTGTTTTTG-3′ located at the *Oxt*^Cre^ allele. The Z/AP was genotyped by performing mouse tails X-gal staining as described in Chen et al. (2011). In brief, tail samples were put in X-gal stock solution (1X PB + 0.02% Triton-100 + 2 mM MgCl_2_ and dissolved in 1 ml dimethylformamide; stock concentration: 100 mg/ml) to a final concentration of 1 mg/ml with X-gal staining buffer [5 mM K_3_Fe(CN)_6_ and 5 mM K_4_Fe(CN)_6_] for 20 min. Seven 8-week-old sexually naïve mice, including three males and four females, were used for the tracing experiments. Three 5-week-old sexually naïve mice were used for double-staining. All animals were generated and bred in the Animal House at the Department of Life Science, National Taiwan University, Taipei, Taiwan. The animals were housed under standard laboratory conditions and maintained on a 12:12-h light/dark cycle and had free access to chow diet and water. The animals were acclimatized to laboratory conditions before the experiment. The experimental protocol was approved by the Institutional Animal Care and Use Committee of the National Taiwan University, Taipei, Taiwan, and conducted according to the National Laboratory Animal Center Guidelines for the use and care of experimental animals.

### Alkaline Phosphatase Staining

Alkaline phosphatase staining followed the protocols outlined in Chen et al. (2011). In brief, adult naïve mice were perfused with PBS for 3 min and with 50 ml of 4% paraformaldehyde (PFA) for 13∼15 min. The brain was dissected and post-fixed with 4% PFA overnight at room temperature. The whole brain in PBS was heat-inactivated in 60°C water bath for 1 h to deactivate endogenous AP. The brain was sectioned at 200 μm thick using a vibratome, and the brain slices were incubated in NBT/BCIP buffer (NBT/BCIP Ready-to-Use Tablets, Roche^®^; one tablet dissolved in 10 ml ddH_2_O) on a shaker (22°C) up to 16 h. The brain slices were washed three times in PBS + 0.1% Tween-20 for 20 min and dehydrated in the following ethanol series—50, 75, 85, and 95% ethanol for 20 min—and finally left in 100% ethanol overnight. Tissues were cleared by incubating in benzyl benzoate (Sigma-Aldrich, B6630)/benzyl alcohol (Sigma-Aldrich, 402834) (BB/BA, 2:1, v/v) for at least 3 min, and slides were mounted with BB/BA. For double-staining, the whole brain in PBS was heat-inactivated in 60°C water bath for 1 h, and the brain slices were sectioned at 80-μm thickness. After incubation with NBT/BCIP buffer on a shaker for 1∼4 h under room temperature, the brain slices were washed three times in PBS + 0.1% Tween-20 for 20 min. The brain slices were incubated with a blocking solution of 0.2% Triton and 6% goat serum in 1X PBS for at least 2 h under room temperature. After blocking, the brain slices were incubated with rabbit anti-oxytocin antibody (1:5,000, immuno-star #20068) at 4°C overnight, washed three times in PBS, and incubated with goat anti-rabbit Alexa-568 (1:500, Invitrogen #A-21069) for 2 h under room temperature. The brain slices were washed three times in PBS and dehydrated in the following ethanol series—25, 50, 75, 85, and 95% ethanol—for 20 min, and finally left in 100% ethanol overnight. Tissues were cleared by incubating in benzyl BB/BA (2:1 v/v) for at least 3 min, and slides were mounted with BB/BA.

### Analysis

The whole mouse brain section template figures (Keith B. J. Franklin and George Paxinos) were used and modified with software (Adobe Illustrator and Photoshop). All images were taken with a Zeiss Z1 inverted fluorescent microscope.

## Results

### Labeling Oxytocin Neurons With Cre and Reporter Mouse

To investigate the oxytocin neuron innervation patterns in the whole mouse brain, we crossed oxytocin-Cre mouse with Z/AP mouse. The Cre recombinase expression is under the control of the endogenous oxytocin locus, which allows oxytocin-expressing neurons to produce human placental AP. We then performed AP staining using Oxt^Cre/+^; Z/AP double-heterozygous mice with both genders (male *n* = 3, female *n* = 4). Since it has been shown that lactating female rats have different oxytocin neuron innervation patterns compared to naïve rats (Knobloch et al., 2012), in this study, we use only naïve male and female mice.

**FIGURE 1:**
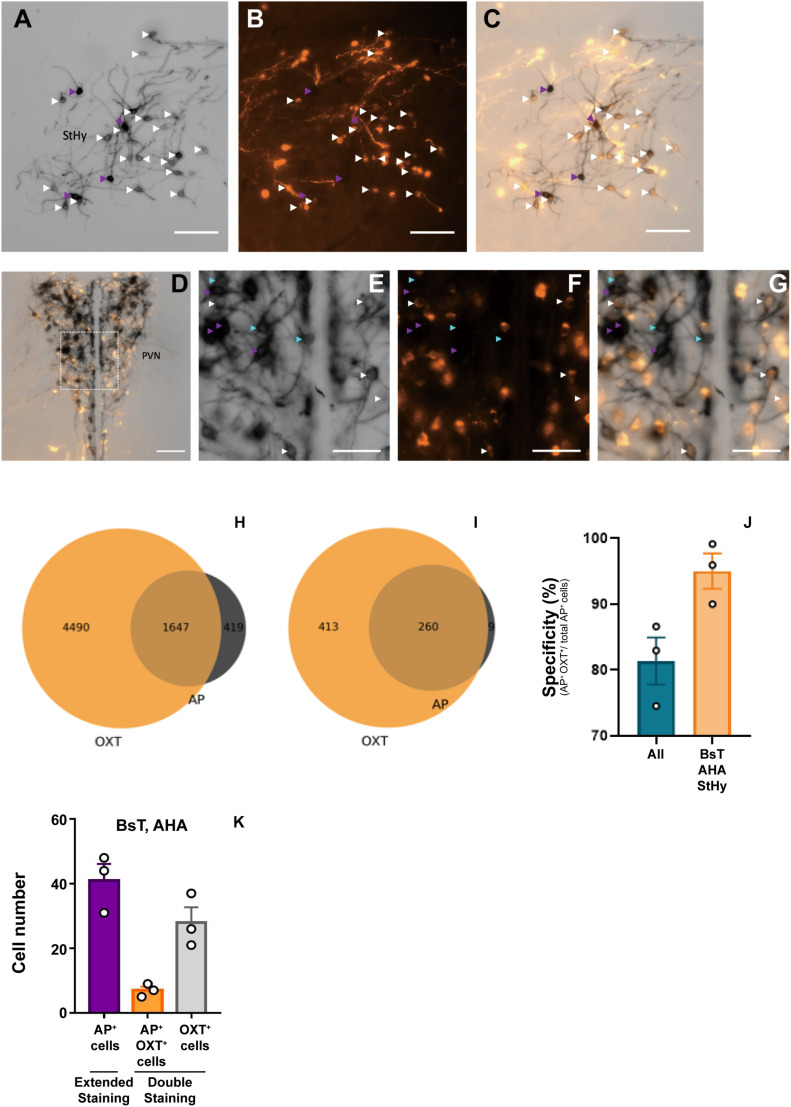
Double-labeling of oxytocin and alkaline phosphatase (AP). **(A–C)** Representative images of colorimetric alkaline phosphatase **(A)** and oxytocin immunofluorescence **(B)** double-staining from OXT^Cre/+^; Z/AP mice brain slice at the StHy. **(D)** Representative images of double-staining at the paraventricular nucleus (PVN). **(E–G)** High magnification of PVN showing colorimetric alkaline phosphatase **(E)**, oxytocin immunofluorescence **(F)**, and merged **(G)** images from **(D)**. Cyan arrowheads indicate AP-positive oxytocin immune-negative cell bodies. White arrowheads indicate co-labeled cell bodies. Purple arrowheads indicate over-saturated AP-staining cells, which were not included for further analysis. **(H)** Venn diagram of double-staining cell count combined from 3 OXT^Cre/+^; Z/AP mice. **(I)** Venn diagram of double-staining cell count combined from 3 OXT^Cre/+^; Z/AP mice from clear-stained areas including the StHy, BsT, and AHA. **(J)** AP staining specificity is calculated from all brain regions or clear-stained areas including the StHy, BsT, and AHA. **(K)** Cell counts from AP staining with extended reaction time or double-staining with short reaction time. *n* = 3. The scale bars in **(A–D)** are 100 μm and **(E–G)** are 50 μm.

To verify the labeling specificity of the Oxt^Cre/+^; Z/AP double-heterozygous mice, we performed double-staining using colorimetric AP staining and oxytocin immunofluorescence staining (Figures 1A–G). Because the dark purple staining from NBT/BCIP buffer could block the fluorescence signal, we stopped the reaction at around 1 h. Under this condition, less than 30% of oxytocin immune-positive cells co-stained with AP (Figures 1H,I). At clear AP-stained regions such as the BsT, StHy, and AHA, about 95% of cell bodies with AP staining are co-labeled with oxytocin antibody staining (Figure 1J). However, at dense cell body regions such as SON and PVN, some AP-positive cell bodies do not co-label with oxytocin. Overall, about 81% of AP-stained cell bodies were co-labeled with oxytocin (Figure 1J). Next, to estimate the labeling sensitivity of AP staining under an extended reaction time (16 h), we calculated the number of AP-labeled cell body in the AHA and BsT, which have a relatively low cell body density for reliable counting. Although we could not verify the staining sensitivity at the PVN and SON, the number of labeled cell bodies from AP staining with extended reaction time is comparable with that from oxytocin immunofluorescence staining at the AHA and BsT (Figure 1K). Therefore, the 30% labeling sensitivity in double-staining may be due to AP under-staining with short reaction time. The actual sensitivity with extended reaction time should be much higher than 30% and could label most oxytocin neurons. These results together suggest that the labeling specificity and the sensitivity of the Oxt^Cre/+^; Z/AP mice were sufficient to target the majority of oxytocin neurons.

**FIGURE 2:**
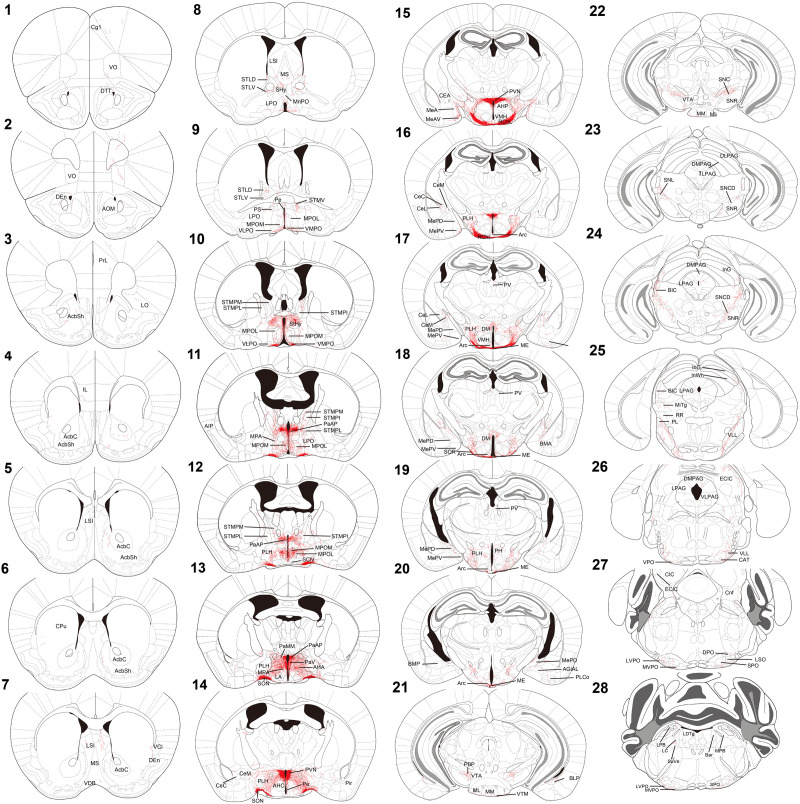
Chartings of schematic coronal sections summarizing the distribution of alkaline-phosphatase-labeled fibers. (1–28) Mouse coronal brain sections, rostrally to caudally. Red color indicates neuron tracing including cell bodies and neurites. Maps are modified from the Franklin and Paxinos (2013) mouse brain atlas. For the abbreviations, see Supplementary Table 1.

For innervation pattern, we performed AP staining protocol with extended reaction time to label fine axon terminals. The level of stained neuron or fiber density within each specific brain region in Table 1, including the olfactory area, the isocortex, the cerebral nuclei, the pallidum, the hypothalamus, the thalamus, the midbrain, the hindbrain, and the medulla. AP-stained cell bodies are exclusively observed in the hypothalamus, the edge of medial amygdala (MeA) area, and the bed nucleus of stria terminalis BST from all seven mice. The innervation pattern is provided for the whole brain in Figure 2. AP staining allows us to cut brain slices with 200-μm thickness to image fibers of oxytocin neuron with minimum disruption. To show full axon structure in the thick brain slice, we used ImageJ to make Z-stack stack images for visualization unless indicated otherwise. Example Z-stack movies were shown as Supplementary Movies.

**TABLE 1:** Oxytocin neuron innervation in mouse brain.

**Area (region)**	**Fibers**	**Individual variance**	**Cell body**
Olfactory area			
AO, anterior olfactory area	±	‡	
TT, tenia tecta	+	‡	
Isocortex			
PrL, prelimbic area	+	‡	
Cg, cingulate cortex	+	‡	
ORB, orbital area	+	‡	
Insular cortex	±	‡	
Pir, piriform cortex	±		
Cerebral nuclei			
Cpu, caudate putamen (striatum)	±	‡	
AcBC, nucleus accumbens core	±		
AcBSh, nucleus accumbens shell	+		
LSI, lateral septal nucleus	++		
MeA, medial amygdala nucleus	+		
CEA, central amygdala nucleus	++		
Pallidum			
BsT, bed nuclei of the stria terminalis	++		§
DB, diagonal band	+++		
Hypothalamus			
LPO, lateral preoptic area	++		
MPO, medial preoptic area	+++		§
StHy, striohypothalamic nucleus	++		§
VLPO/VMPO, ventral lateral/medial preoptic nucleus	++++		§
SHy, septohypothalamic nucleus	+++		§
PS, parastrial nucleus	++		
AHA, anterior hypothal area, anterior part	+++		§
Arc, arcuate hypothalamic nucleus	+++		
PVN, paraventricular nucleus of hypothalamus	+++++		§
Pe, periventricular hypothalamic nucleus	++		§
SON, supraoptic nucleus	+++++		§
SOR, retrochiasmatic supraoptic nucleus	+++		§
PLH, peduncular part lateral hypothalamus	++++		
Thalamus			
PV, paraventricular nucleus of thalamus	+++	‡	
Midbrain			
LPB, lateral parabrachial nucleus	+		
LC, locus coeruleus	++	‡	
Bar, Barrington’s nucleus	±	‡	
VTA, ventral tegmental area	±		
SNC, substantia nigra compact part	++		
PAG, periaqueductal gray	++		
IC, inferior colliculus	++		
Hindbrain			
MY-mot, medulla, motor related	++		
Spinal cord	+		

*Fiber intensity is classified with: extremely dense (+++++); very dense (++++); dense (+++); medium (++); few branches/fibers (+); sparse (±); no fiber (−). Individual variance observed (‡). Cell bodies observed (§).*

### Oxytocin Neurons in the Hypothalamic Area

**FIGURE 3:**
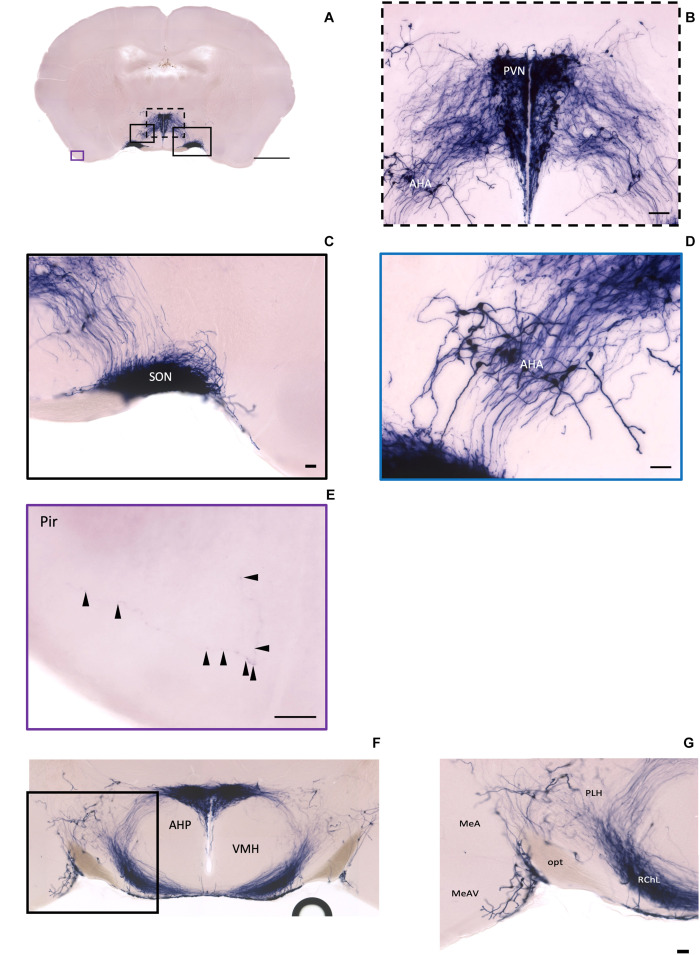
Oxt neurons in the hypothalamus area and the piriform cortex. **(A)** Overview of alkaline phosphatase (AP)-staining whole coronal section. Scale bar = 1 mm. **(B)** Cell bodies and neurites in the PVN and the AHA regions. Scale bar = 100 μm. **(C)** Dense cell bodies in the SON. **(D)** Medium density of cell bodies located in the AHA. **(E)** Sparse projection fibers in the Pir; arrowheads indicate the location of the fibers. Scale bar = 50 μm. **(F,G)** The coronal section overview with the posterior part of the PVN and the axon bundles toward the ME pass-through lateral hypothalamus RChL. Scale bar = 50 μm. PVN, paraventricular nucleus, SON, supraoptic nucleus; AHA, anterior hypothalamic area; Pir, piriform cortex; RChL, retrochiasmatic nucleus, lateral part; PLH, peduncular part of lateral hypothalamus; AHP, anterior hypothalamic area, posterior part; MeA, medial amygdaloid nucleus anterior part; MeAV, medial amygdaloid nucleus anteroventral part.

**FIGURE 4:**
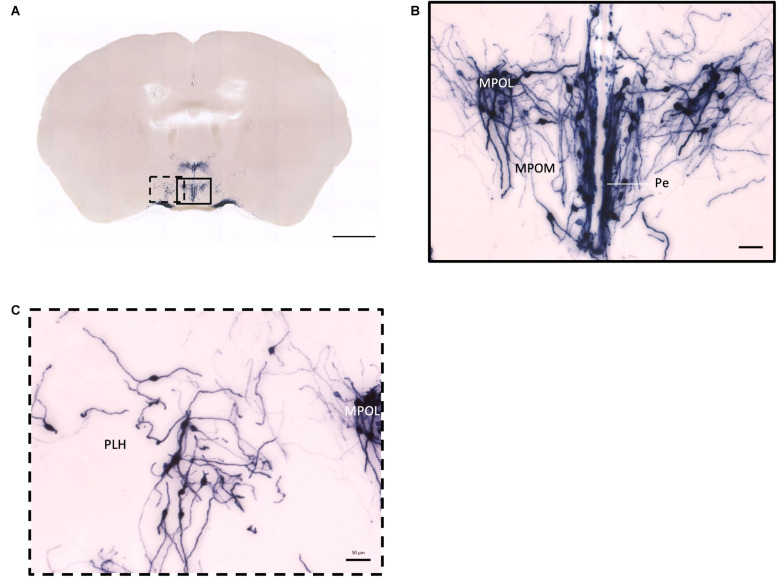
Projection of OXT neurons in the preoptic area. **(A)** Overview of alkaline phosphatase-staining whole coronal section. Scale = 1 mm. **(B)** Labeled cell bodies and neurites locate in the MPOL, MPOM, and Pe near the 3V. **(C)** Labeled cell bodies in the PLH area. Scale = 50 μm. MPOL, medial preoptic nucleus lateral part; MPOM, medial preoptic nucleus medial part; Pe, periventricular nucleus; PLH, peduncular part lateral hypothalamus.

**FIGURE 5:**
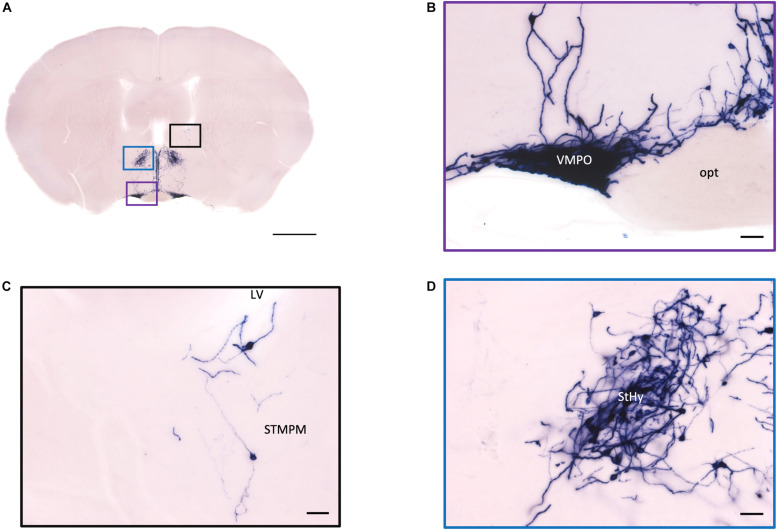
Projection of OXT neurons in the preoptic area, the pallidum, and the striohypothalamic nucleus. **(A)** Overview of AP-staining whole coronal section. Scale = 1 mm. **(B)** Very dense cell bodies and neurites in the VMPO near the optic tract. Scale bar = 50 μm. **(C)** Few cell bodies located in the STMPM close to the LV. **(D)** Dense cell bodies in the StHy. VMPO, ventromedial preoptic nucleus; opt, optic tract; STMPM, bed nucleus of the stria terminalis medial division, ventral part; LV, lateral ventricle; StHy, striohypothalamic nucleus.

**FIGURE 6:**
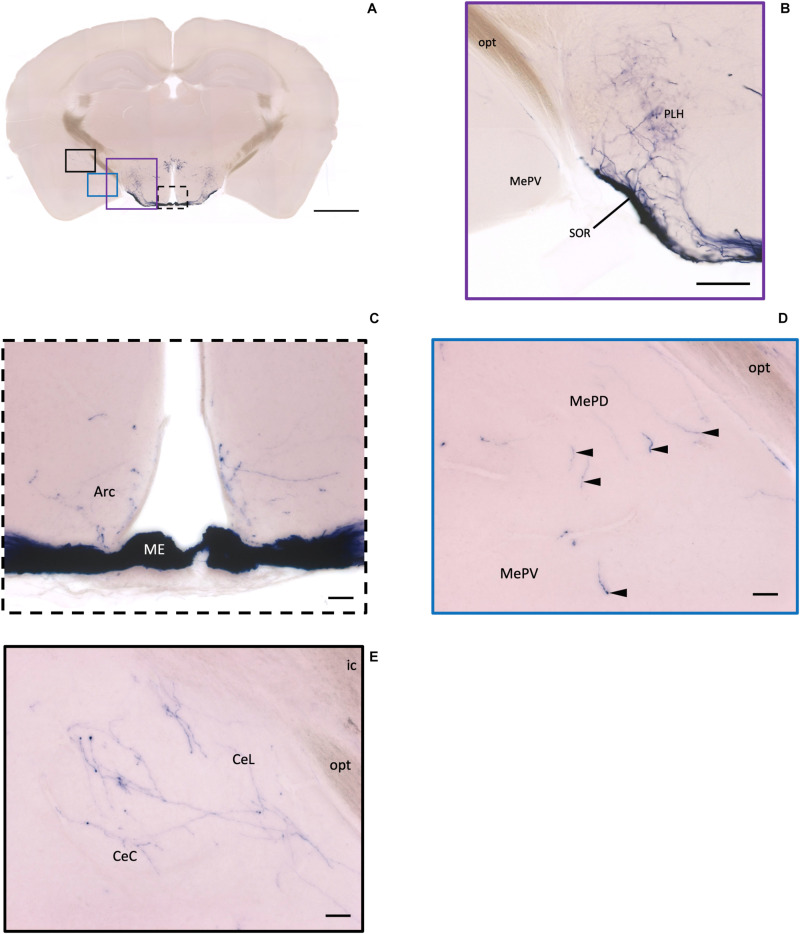
Projection of OXT neurons in the arcuate hypothalamic nucleus and the amygdala subdivisions. **(A)** Overview of alkaline phosphatase-staining whole coronal section. Scale = 1 mm. **(B)** Some thin axon fibers with puncta structure in the PLH. **(C)** Sparse OXT neuron branches in the Arc and dense axon bundle in the ME. **(D)** Few pass-through fibers (arrowheads) in the MeA subregions. **(E)** Medium density of OXT neuron branches in the CeM subregions. Scale bar = 50 μm. Arc, arcuate hypothalamic nucleus; PLH, peduncular part lateral hypothalamus; SOR, retrochiasmatic supraoptic nucleus; ME, median eminence; MePD, medial amygdala posterodorsal part; MePV, medial amygdala posteroventral part; CeL, central amygdala capsular part; CeM, central amygdala medial division.

**FIGURE 7:**
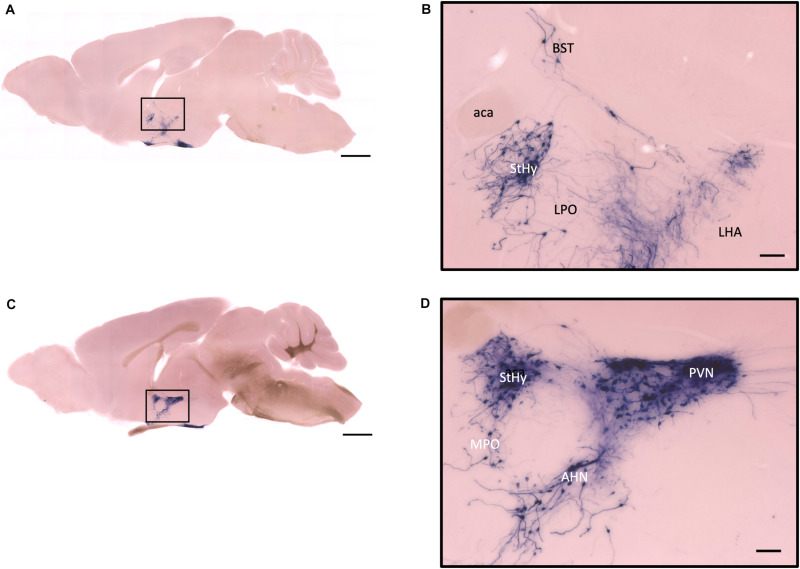
Sagittal view of OXT neuron projection. **(A,C)** Two sagittal AP staining brain sections from a female mouse. Scale bar = 1 mm. **(B,D)** Large magnification of images from **(A,C)**, showing OXT neurons and their fibers in the hypothalamic area and the preoptic area. Scale bar = 100 μm. BST, bed nucleus of the stria terminalis; aca, anterior commissure, anterior part; StHy, striohypothalamic nucleus; LPO, lateral preoptic area; LHA, lateral hypothalamic area; MPO, medial preoptic area; AHN, anterior hypothalamic nucleus; PVN, paraventricular nucleus.

In the mouse hypothalamus, the PVN and SON are two primary regions which release oxytocin. It has been shown that oxytocin-releasing neurons can be divided into parvocellular and magnocellular cells depending on their morphological and electrophysiological characteristics. However, since AP staining labels all neurites from the dense cluster of OXT neurons in both the PVN and SON, we could not identify the subtypes of OXT neurons and the number of cell bodies within these two nuclei morphologically. In the anterior PVN, most cell bodies are located near the dorsal part of the third ventricle (3V) (Figures 3A,B). Some neurons and their axons extended, along with the 3V, ventrally and rostrally to the periventricular hypothalamic nucleus (Pe) (Figures 4A,B). Although these axons extended to the dorsal edge of the anterior suprachiasmatic nucleus (SCN), there is no AP staining observed inside the SCN (Figure 3A). In the SON, the cell bodies of OXT neurons are densely located laterally to the optic tract (Figure 3C). There are many passing axon fibers between the PVN and SON through the anterior area of the anterior hypothalamus (AHA). In addition, some cell bodies are labeled in the AHA (Figure 3D) and the peduncular part of the lateral hypothalamus (PLH) (Figure 4C). Few cell bodies and their dendrites extend to the edge of the MeA along the ventral side of the optic tract (Figures 3F,G and Supplementary Movie 1). In the POA, the medial preoptic nucleus (MPO), including the caudal lateral part of the MPO (MPOL) and the medial part of the MPO (MPOM) (Figure 4B and Supplementary Movie 2), was labeled with the lower density of cell bodies compared to the PVN and SON. However, the cell body density in the ventral medial preoptic nucleus (VMPO) is relatively high (Figures 5A,B and 9D), similar to the SON and PVN. The striohypothalamic nucleus (StHy) in the rostral POA shows many labeled cell bodies (Figure 5D). The retrochiasmatic supraoptic nucleus (SOR) is the most caudal brain region with few oxytocin-labeled cell bodies (Figures 6A,B). There is no cell body labeled rostral to the StHy (Figure 7).

### Fibers of Oxytocin Neurons in the Cortex and Olfactory Areas

**FIGURE 8:**
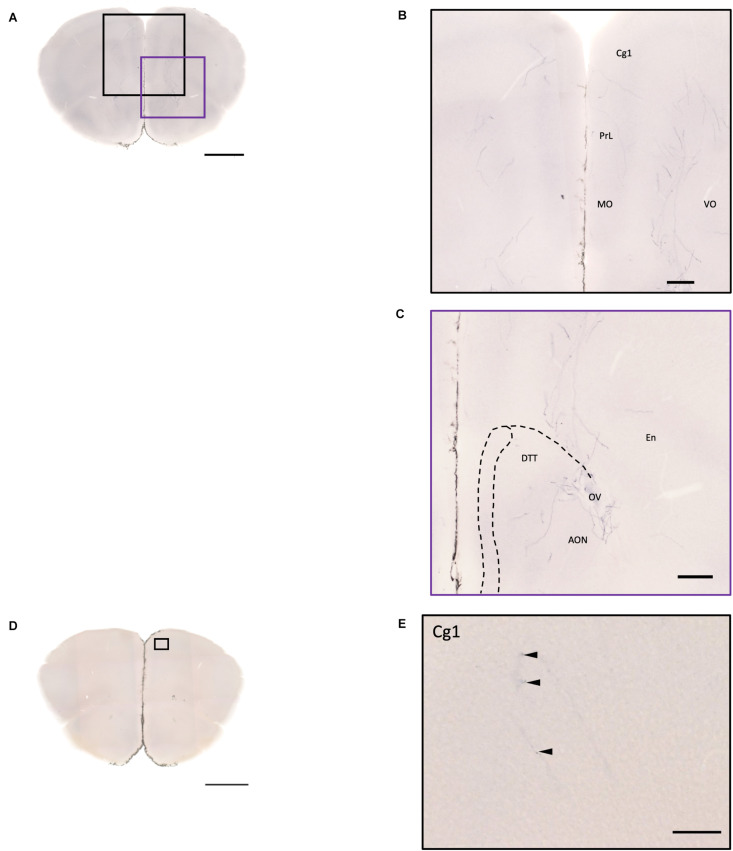
Projection of OXT neurons in the prefrontal brain area. **(A,D)** Overview of alkaline phosphatase-staining whole coronal section. Scale bar = 1 mm. **(B)** Innervation of oxytocin neurons in the prefrontal cortex such as the Cg, the PrL, and part of the orbital cortex. **(C)** Innervation of oxytocin neurons in the DTT and anterior olfactory nucleus. Scale bar = 200 μm. **(E)** Single fiber of oxytocin neuron with some enlarged varicosities (arrowheads) in the anterior cingulate cortex. Scale bar = 100 μm. Cg, cingulate cortex; DTT, dorsal tenia tecta; PrL, prelimbic cortex; MO, medial orbital cortex; VO, ventral orbital cortex; OV, olfactory ventricle; En, endopiriform nucleus.

**FIGURE 9:**
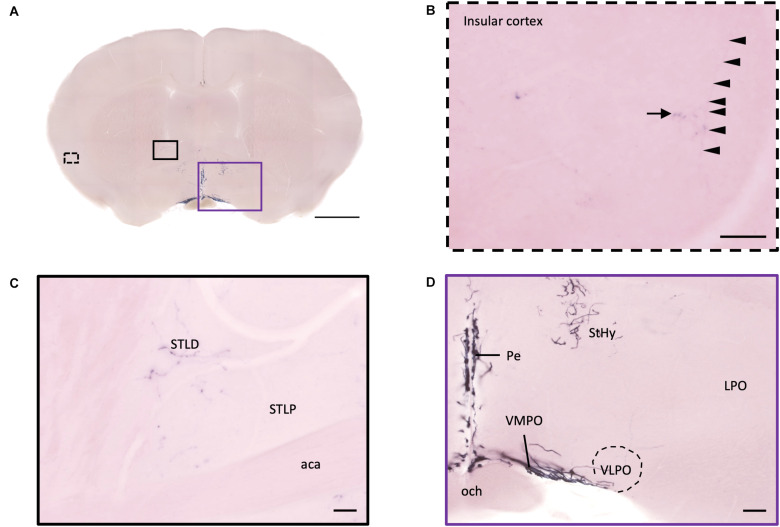
Projection of OXT neurons in the bed nucleus of stria terminalis, preoptic area, and insular cortex. **(A)** Overview of alkaline phosphatase-staining whole coronal section. Scale bar = 1 mm. **(B)** Single fiber (arrowheads) with terminal-liked branches (arrow) in the insular cortex. **(C)** Sparse oxytocin neuron terminals in the STLD and the STLP near the caudate putamen. Scale bar = 50 μm. **(D)** Neurites of oxytocin neuron in the VMPO region near the VLPO. Scale bar = 100 μm. STLD, bed nucleus of the stria terminalis lateral division, dorsal part; STLP, bed nucleus of the stria terminalis lateral division, posterior part; aca, anterior commissure, anterior part; StHy, striohypothalamic nucleus; VMPO, ventromedial preoptic nucleus; VLPO, ventrolateral preoptic nucleus; LPO, lateral preoptic area; Pe, periventricular hypothalamic nucleus; och, optic chiasm.

In the isocortex and olfactory area, some sparse axons can be observed in the orbital cortex, insular area, AON, TT, and piriform cortex (Pir). There are obvious branching terminals in the AON, TT, Pir, and orbital cortex for most animals. In the orbital cortex (OC), we can observe both pass-through and branching axons. Most branching axons are located between the ventral orbital cortex (VO) and the medial orbital cortex (MO) (Figures 8A,B). In addition, some pass-through axons partially extended to the edge of the forceps minor of the corpus callous (fmi). In the olfactory area, few branching axons can be observed in the AON, while pass-through axons can be observed in the dorsal part of the tenia tecta (DTT) (Figure 8C). Some axons could be observed in the cingulate cortex (Figures 8D,E). In the Pir, sparse axons, tracing back from the MeA through the cortical amygdala (CoA) and posterior lateral cortical amygdala (PLCo) regions, can be observed in both genders. Interestingly, although axons in the Pir do not show elaborate terminal structure (Figure 3E and Supplementary Movie 3), they are much complex than straight pass-through axons in the CoA and PLCo. Finally, we only observed one male individual with very sparse and loose innervation at the insular cortex areas (Figures 9A,B).

### Interbrain Pallidum and Thalamic Area

**FIGURE 10:**
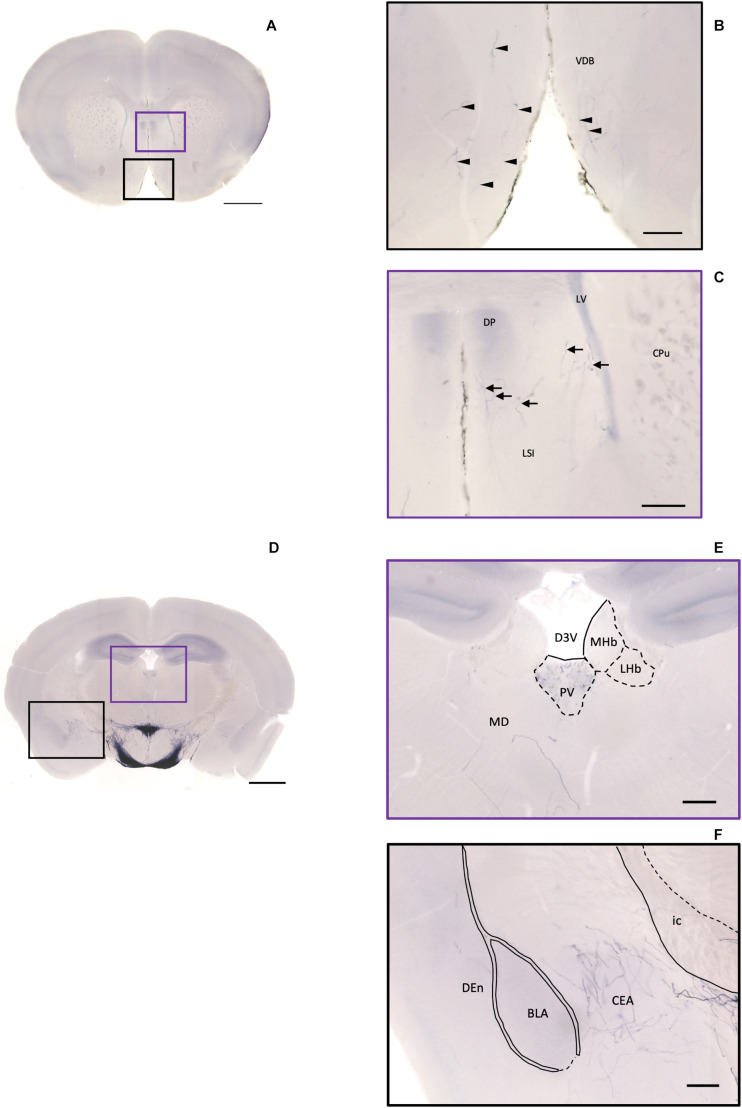
Projection of OXT neurons in the diagonal band, the septal nucleus, the thalamus, and the amygdala. **(A)** Overview of alkaline phosphatase (AP)-staining whole coronal section. Scale bar = 1 mm. **(B)** Oxytocin neuron fibers in the VDB (arrowheads). **(C)** Some terminal-like branches from oxytocin neuron in the LSI (arrows). Scale bar = 200 μm. VDB, nucleus of the vertical limb of the diagonal band; LSI, lateral septal nucleus, intermediate part; CPu, caudate putamen; DP, dorsal peduncular cortex; LV, lateral ventricle. **(D)** Overview of AP-staining whole coronal section. Scale bar = 1 mm. **(E)** Oxytocin neurons innervate the PV near the habenular nucleus. **(F)** Many terminal branches of oxytocin neuron in the central amygdala medial division and few pass-through fibers in the DEn. Scale bars = 200 μm. D3V, dorsal third ventricle; PV, paraventricular nucleus of thalamus; MHb, medial habenular nucleus; LHb, lateral habenular nucleus; MD, thalamic nucleus; CEA, central amygdaloid nucleus; BLA, basolateral amygdaloid nucleus, anterior part; Den, dorsal endopiriform claustrum; ic, internal capsule.

In the pallidum, the medial septal nucleus (MS) and the ventral diagonal band nucleus (VDB) have few pass-through axon fibers that seem to innervate the nucleus accumbens or olfactory area (Figures 10A,B). In the bed nucleus of the stria terminal BST, few cell bodies and some sparse thin axon fibers can be observed close to the lateral ventricle located at the posterior part of the bed nucleus of the stria terminal medial division (STMPM) (Figure 5C). Few sparse axons can be observed rostrally in the bed nucleus of the stria terminal lateral division STL (Figure 9C). In the thalamic area, the PV has few sparse and thin axon terminals. Interestingly, the density of terminal fibers shows high individual variability, with some individuals having barely detectable terminals (Figures 10D,E).

### Striatum

**FIGURE 11:**
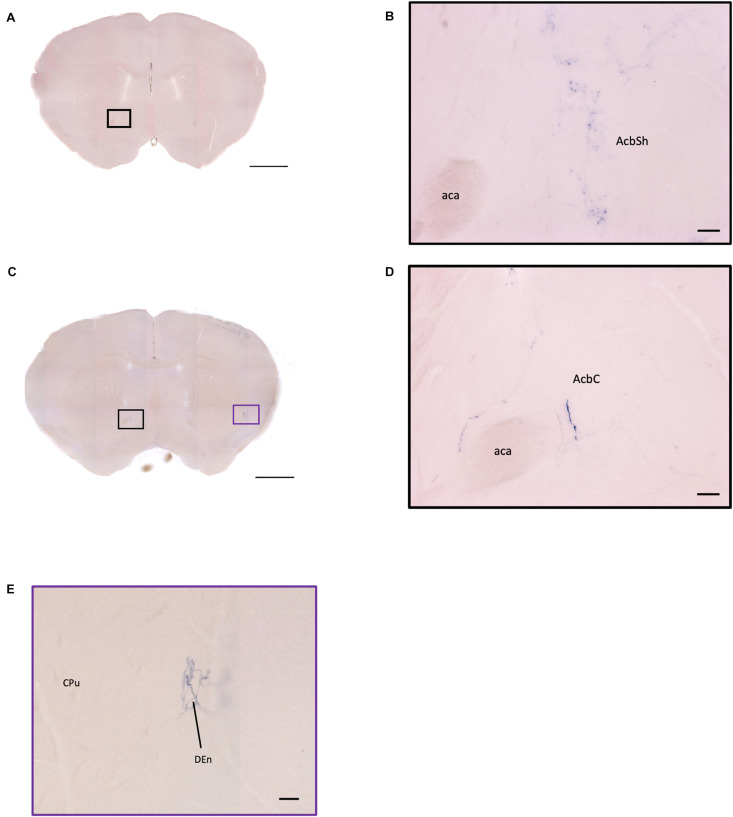
OXT neuron innervation in the nucleus accumbens subregions. **(A,C)** Overview of AP-staining whole coronal section. Scale bar = 1 mm. **(B)** Sparse terminals of oxytocin neurons in the AcbSh. Scale bar = 50 μm. **(D)** Few fibers of oxytocin neuron in the AcbC. Scale bar = 50 μm. **(E)** Terminal-like plexus from oxytocin neuron in the DEn. Scale bar = 100 μm. AcbSh, nucleus accumbens, shell; AcbC, nucleus accumbens, core; aca, anterior commissure, anterior part; DEn, dorsal endopiriform claustrum; CPu, caudate putamen.

**FIGURE 12:**
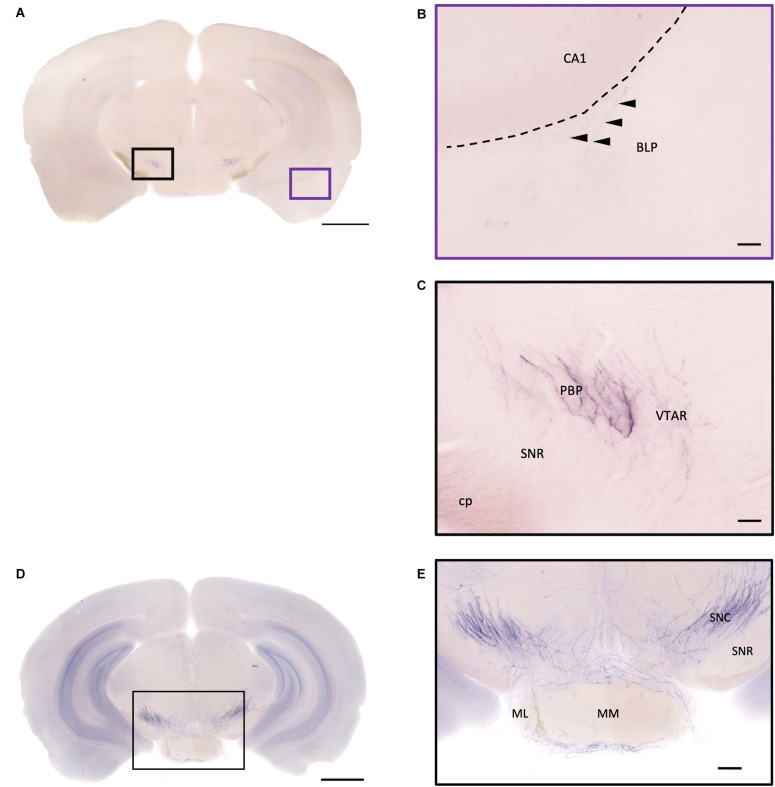
Projection of OXT neurons in the hypothalamic mammillary nucleus and the midbrain area. **(A,D)** Overview of alkaline phosphatase-staining whole coronal section. Scale bar = 1 mm. **(B)** The sparse terminal-like fiber in the BLP (arrowheads), near the ventral part of the CA1 region. Scale bar = 50 μm. **(C)** Some heavily stained fibers and plexus of branches from oxytocin neuron in the PBP. The VTAR also has some terminal-like branches, while the SNR seems to lack innervation. **(E)** Many oxytocin neuron fibers in the SNC. Some branches form a shell-like innervation pattern at ML. Scale bar = 50 μm. MM, medial mammillary nucleus; ML, medial mammillary nucleus lateral; SNC, substantia nigra compact part; SNR, substantia nigra reticular part; BLP, basolateral amygdala nucleus posterior part; CA1, field CA1 hippocampus; cp, cerebral peduncle; PBP, parabrachial pigmented nucleus of the VTA; VTAR, ventral tegmental area.

In the striatum, many pass-through axons can be observed in the lateral septal nucleus intermedial part (LSI) (Figure 10C). These axons seem to project to the AcB and prefrontal cortex area. In the nucleus accumbens, the shell region has many elaborated branches (Figures 11A,B), while the core region has relatively few branches (Figures 11C,D). However, due to the lack of core and shell landmarks, we cannot completely be sure whether these few branches in the core region are specifically targeting the core or if they are at the boundary of the shell region. In the endopiriform nucleus (En), few sparsely labeled axon branches can be observed elongating from the rostral to the caudal region (Figures 10F, 11E). In the amygdala, the MeA has many cell bodies with elaborate dendritic branches (Figures 3F,G). Although many axons travel through the MeA posterior, dorsal, and ventral part (MePD, MePV) to the central amygdala and Pir, no axon terminal-like neurites are observed in the MeA (Figure 6D). In the central amygdala, many axon terminal branches cover the whole central amygdala region including the central amygdala medial division (CeM), central amygdala lateral division (CeL) and central amygdala capsular part (CeC) (Figures 6E, 10F). Some axons near the lateral ventricle project further caudally to the basolateral amygdala posterior region (BLP) (Figures 12A,B). Unlike the central amygdala and BLA, we only observe single axon-like structures without branches in the BLP region.

### Fibers of Oxytocin Neurons in the Hypothalamus Region

At the hypothalamic area, although few cell bodies can be observed in the PLH and AHA region, lots of axons pass through these two regions between the PVN and SON (Figures 3B–D). In contrast, there are almost no axon terminals in the ventral medial hypothalamic nucleus (VMH) and the posterior area of the anterior hypothalamic (AHP) (Figure 3F). Although most of the VMH has no oxytocin fiber, some passing axons may go through the ventrolateral part of the VMH (Figure 3F). Ventrally to the medial hypothalamus region, very dense passing axon fibers at the retrochiasmatic area (RCh) (Figure 3F) and the median eminence (ME) are labeled (Figures 6A,C). There are some axon terminals in the arcuate hypothalamus nucleus (Arc) (Figure 6C and Supplementary Movie 4). Many axon fibers with puncta-like structure could be observed in the lateral hypothalamus (PLH), while some passing-through axons could be observed in the medial tuberal nucleus (MTu) (Figure 6B). Further caudally at the ventral part of the hypothalamus, axons and branching terminals can be observed covering the mammillary nucleus. However, the central part of the medial mammillary nucleus (MM) and the lateral mammillary nucleus (LM) have almost no fibers. Most branches cover the anterior, posterior, ventral, and dorsal edges of the mammillary nucleus to form a shell-like pattern (Figures 12D,E).

### Midbrain

**FIGURE 13:**
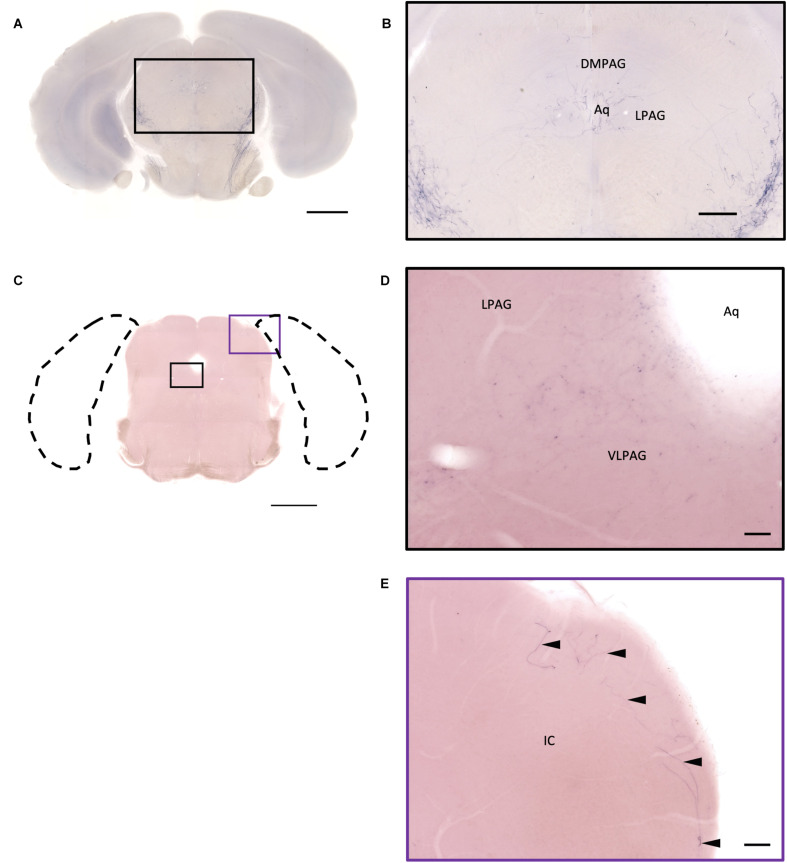
Projection of OXT neurons in the midbrain. **(A,C)** Overview of alkaline phosphatase (AP)-staining whole coronal section. Scale bar = 1 mm. **(B)** Oxytocin neuron innervation in the LPAG and the DMPAG near the Aq. Scale bar = 300 μm. **(D)** Many terminal-like branches in the VLPAG. Scale bar = 50 μm. **(E)** AP-staining branches and fibers (arrowheads) at the edge of the ECIC. Scale bar = 100 μm. DMPAG, dorsomedial periaqueductal gray; LPAG, lateral periaqueductal gray; ECIC, inferior colliculus external nucleus; Aq, aqueduct.

In the midbrain, the rostral part, VTA, and parabrachial pigmented nucleus (PBP) have some thicker pass-through fibers and thinner terminal branches (Figure 12C). Unlike the VTA and PBP which are covered with terminals and axons, the edge of the SN has many pass-through fibers (Figure 12C). At the posterior part of the substantia nigra pars compact (SNc), some terminals could be observed (Figure 12E). Further caudally, the axon bundles project both dorsally and ventrally to the PAG and spinal cord, respectively. The dorsal bundles enter the PAG and sparse terminal fibers extend from the ventral lateral periaqueductal gray (VLPAG) and lateral periaqueductal gray (LPAG) to the dorsal medial periaqueductal gray (DMPAG) (Figures 13A–D). Many axons pass dorsally through the brachium of the colliculus (BIC), and perhaps the intermedial gray of the superior colliculus (InG) and the intermediate white layer of the superior colliculus (InWh), and finally form branches at the inferior colliculus (IC) (Figure 13E). For the ventrally projecting axon bundles, we can observe fiber tracts passing through the ventral lateral lemniscus (VLL), paralemniscal nucleus (PL), and nucleus central acoustic stria (CAT) into the hindbrain (Figure 2, slide 25 and 26).

### Hindbrain and Medulla

**FIGURE 14:**
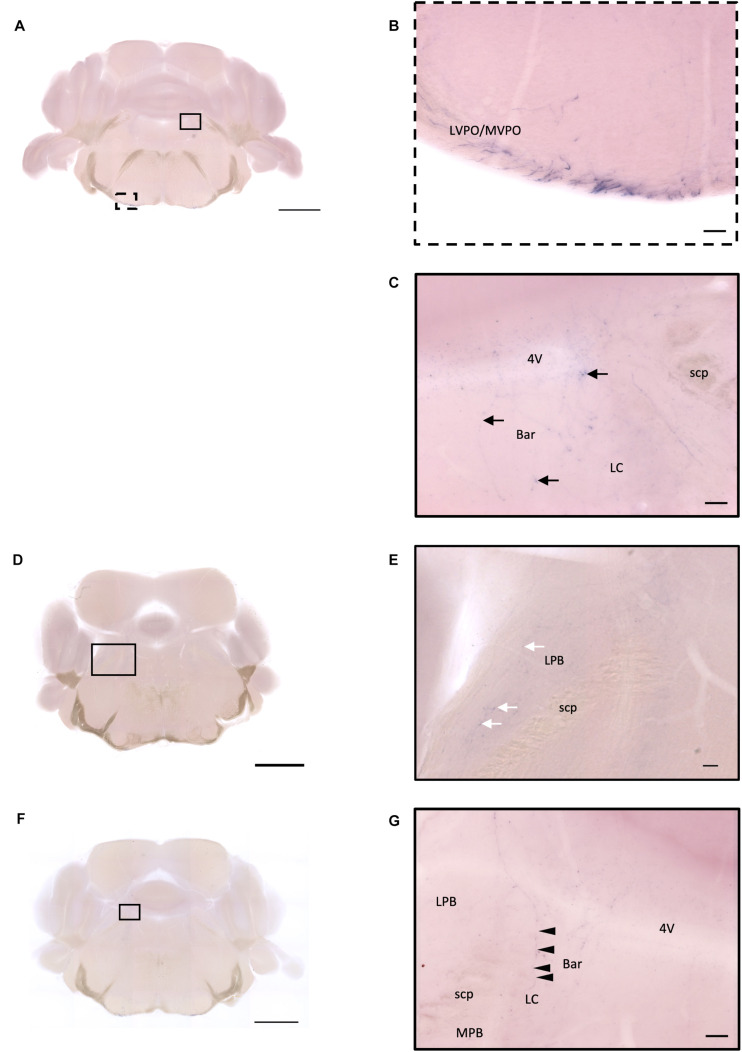
Projection of OXT neurons in the hindbrain and medulla. **(A,D,F)** Overview of alkaline phosphatase (AP)-staining whole coronal section. Scale bar = 1 mm. **(B)** Many pass-through fibers in the MVPO and the LVPO. **(C)** Innervation fiber of oxytocin neurons in the Bar (arrows). **(E)** Few AP-staining branches in the LPB (white arrows). **(G)** Innervation fibers of oxytocin neuron in the LC (arrowheads). Scale bar = 50 μm. 4V, fourth ventricle; MVPO, medioventral periolivary nucleus; LVPO, lateroventral periolivary nucleus; Bar, Barrington’s nucleus; LC, locus coeruleus; scp, superior cerebellar peduncle; LBP, lateral parabrachial nucleus; MPB, medial parabrachial nucleus.

In the ventral part of the hindbrain, many fibers pass through the preolivary region (POR), near the lateroventral preolivary nucleus (LVPO) and medial–ventral preolivary nucleus (MVPO), to the spinal cord (Figures 14A,B). On the other hand, in the dorsal part of the hindbrain, some terminals could be observed in Barrington’s nucleus (BAR) (Figures 14A,C) and the lateral regions of the parabrachial nucleus (LPB) (Figures 14D,E). In addition, few branching fibers can be observed at the edge of the LC (Figures 14F,G), although the apparent fiber density is much lesser than that at the LPB.

## Discussion

### Comparison to Previous Reports

Here we report the innervation pattern of mouse oxytocin neurons and their potential target sites in the whole brain using genetic reporters. In previous literature, immune-histochemistry using antibodies for oxytocin or neurophysins was the primary method to label oxytocin neurons and their fibers (Sofroniew, 1980, 1983a,b; Rhodes et al., 1981; Sawchenko and Swanson, 1982; Knobloch et al., 2012). Recently, injection of virus reporters combined with OXT-Cre mice were used to map the innervation pattern of the PVN and SON (Marlin et al., 2015; Yao et al., 2017). The immune-histochemistry relies on the specificity of the antibody. However, the sensitivity is relatively low compared to that of a genetic reporter. Using virus reporters with OXT-Cre can increase the labeling intensity of oxytocin-expressing neurons, enhancing the contrast to observe the fine structure of oxytocin neurons. However, the virus vector might not label all neurons, and the injection may injure some neurons and their axon fibers during surgery. By using the OXT-Cre and AP reporter line, we were able to label oxytocin-releasing neurons in the whole brain. This method provides high labeling contrast and specificity compared to immune-staining and virus/dye injection, respectively. In addition, with an endogenous reporter system, we can reduce potential surgery complications to faithfully report the innervation patterns of oxytocin neurons in individual mice. Therefore, this work shows comprehensive projection patterns of the oxytocin neuron in the whole brain, which was separately reported in previous literature. However, colorimetric AP staining has some limitations. First, the bright-field images taken here are hard to reconstruct in 3D. Although individual image at specific focus planes provides high detection contrast, many signals are hard to perceive in stacked images. Second, the prolonged AP staining reaction time (up to 16 h) allows us to observe great details in the axon and dendrite architecture. However, in condensed areas with many cell bodies such as the paraventricular, supraoptic, and preoptic nuclei, the staining is too dense to observe the single neuron and their dendritic structure clearly even with a high-resolution objective lens. Therefore, we could not identify the cell number or differentiate magnocellular and parvocellular cell morphology in these regions. Finally, the Cre-loxP system reports every neuron which expresses oxytocin, even if only briefly, during the development stage. Although previous reports show high specificity of the OXT-Cre mouse that we used in this study, it is still possible that some neurons do not produce oxytocin when we collected brain samples from 3- to 4-month-old mice.

In the previous reports, some studies showed that oxytocin-immunoreactive fibers were present in the ventral hippocampus region of mice (Sofroniew, 1983b; Castel and Morris, 1988), and that some fibers originating from the PVN and SON innervate the CA1, CA2, and CA3 regions in the hippocampus of female rats (Knobloch et al., 2012; Mitre et al., 2016). However, we never observed oxytocin-Cre labeled fibers in the hippocampus subareas in all of our subjects. We only observed some branching fibers in the BLP area near the hippocampus. In addition, a report showed that OXT terminal-like fibers were observed in the auditory cortex, visual cortex, and barrel cortex in virgin female mice (Marlin et al., 2015). In contrast, we did not observe any OXT fiber in these cortical areas in our female mice. However, due to the lack of specific anatomical landmarks for the different cortex areas in mouse, it is also possible that these fibers are targeting the OC. We speculate that the innervation patterns of oxytocin neurons to the cortex or hippocampus area is highly specific to each individual, which may be the result of individual experiences prior to sample collection. Alternatively, this discrepancy may simply derive from the difference between species or mouse stains.

Oxytocin receptor distribution has sexual differences in some brain regions in rodents, and its activation in different brain regions during social stimulus has been investigated (Kim et al., 2015). Previous studies suggested that the projection sites and innervation patterns of oxytocin immune-reactive neurons between male and female do not differ greatly. Here we also compared oxytocin neuron projection sites between three male and four female mice. Although the branching density shows a high variation in some brain nuclei between individuals, we did not observe an apparent innervation pattern variation between the two different genders. Further study with a higher number of animals is required to conclude whether there is a gender difference. Most interestingly, the full innervation pattern in naïve mice suggests that many behavior functions could be modulated by oxytocin before sexual interaction. Alternatively, the sparse axon terminals in many brain regions could extend furthermore during pregnancy or parenting period to enforce parenting behaviors.

### Innervation in the Hypothalamic Area

Our results show a similar pattern of cell bodies and neurites in the hypothalamus compared to previous reports. The hypothalamic area, which plays a versatile role in social, autonomic, and physiological function, contains many fibers, terminals, and cell bodies in various rostral to caudal regions. Although we have a very high level of staining in the PVN and SON, the dense AP-labeled cell bodies and fibers preclude us to decipher the detailed connections around these regions. In addition to the PVN and SON, we consistently observed that AHA, a relay center regulating endocrine, autonomic nerves, feeding behavior, and response to stress (Kavushansky et al., 2016), has a medium level of oxytocin cell bodies, suggesting a potential reciprocal connection between the oxytocin system and feeding behavior or stress response.

The functions of the POA in the hypothalamus are very complicated. It has been shown that POA is a critical region for thermoregulation and energy balance (Boulant, 2000; Yu et al., 2018). Some sub-areas like the MPO region are associated with sexual behavior (Merari and Ginton, 1975; Dominguez and Hull, 2005; Wei et al., 2018). Sex dimorphism of androgen receptor- and estrogen receptor-expressing neurons in the POA has been clearly described (Normandin and Murphy, 2008). Many neurons in the StHy, which receives inputs from the amygdala for sexual or physiological function (Normandin and Murphy, 2008), also express estrogen receptors (Axelson and Leeuwen, 1990). However, we did not observe significant differences in OXT fiber density in these areas. In the medial POA, we observed very dense oxytocin cell bodies located in the MPO regions, while some neurites extended to the MPOM near the 3V. It has been shown that the MPO is associated with sexual and parental behavior, potentially through its connection to the midbrain or other brain areas (Kohl et al., 2018; Wei et al., 2018). Since staining at the MPO region is comparable to the PVN and SON regions, the specific function of oxytocin-expressing neurons in the MPO should be investigated further.

It is well known that the VMH is associated with appetite, feeding, energy balance, and sexual behavior (Griffin and Flanagan-Cato, 2011). Some literature has proposed that innervation of the VMH by oxytocin fibers may regulate female hormone release and sexual behavior in rats (Griffin et al., 2010; Narita et al., 2016). Although we did not observe oxytocin fibers in the VMH regions, some sparse terminals were very close to the ventral part of the VMH subdivision. The oxytocin fibers here may participate in regulating specific functions in the sub-regions of the VMH through indirect pathways or short-distance paracrine pathways.

The MeA, which regulates social recognition (Ferguson et al., 2001; Samuelsen and Meredith, 2011), has been suggested to be one of the target regions for PVN or SON using Cre-dependent GFP reporter virus or anterograde tracing dye (Alonso et al., 1986; Yao et al., 2017). Here we observed few labeled cell bodies and their dendrites in the anterior part of the MeA and some passing fibers in the posterior part of the MeA. Since oxytocin neurons have both somatodendritic and axonal release, it is possible that cell bodies and axons could provide oxytocin input at the anterior and the posterior parts of the MeA, respectively. The CeA and its subdivision, regulating the fear conditioning response in rats, are targeted by the oxytocin neuron from the PVN (Knobloch et al., 2012). Here we observed many terminal branches, which originated from the posterior part of the MeA, covering large areas in the CeA. The oval shape of the CeA can be easily identified by these sparse oxytocin fibers in the coronal sections, suggesting strong interactions between the oxytocin system and the CeA functions. Finally, some fibers from the ventral part of the MeA seem to extend laterally through the basomedial amygdala region to the Pir. However, these fibers only form few simple branches, suggesting that smell-related social behavior regulation by oxytocin may occur primarily in the MeA and CeA regions.

### Innervation in Pallidum and Limbic System

The BST (or called the BNST) part of the forebrain limbic system is a complex relay center which receives signal from the medial prefrontal cortex (mPFC), hippocampus, hypothalamus, and brain stem to regulate neuroendocrine, behavior response, fear learning, and the autonomic system (Wakerley et al., 1998; Ko, 2017; Martinon et al., 2019). In this study, the BST is the only extra-hypothalamic region which has labeled oxytocin cell bodies. The BST regions have some sparse cell bodies and fibers which form terminals close to the lateral and dorsal 3rd ventricle. It is possible that these terminals could release oxytocin into the ventricle fluid or are specifically involved in local circuitry regulation in the STMPM sub-regions of the BST.

The hippocampus is essential for spatial and social memory. It has been shown that oxytocin and oxytocin receptors at CA2 and CA3 regions are involved in the modulation of social memory formation (Kovács et al., 1979; Hitti and Siegelbaum, 2014; Raam et al., 2017; Lin et al., 2018). However, we did not observe any oxytocin fibers in our female virgin mice. It is possible that oxytocin fibers only innervate the hippocampus after pregnancy or giving birth to offspring. We observed some fibers located ventrally to the hippocampus at the posterior BLP region. We speculate that these fibers may be involved in fear response regulation locally in the BLP or are waiting for innervation to the hippocampus after pregnancy or giving birth to offspring.

### Innervation in Basal Ganglia

Previous reports showed contradictory results for the presence of oxytocin fibers in the CPu of the basal ganglia. Some studies showed sparse fibers in different individuals without consistent patterns after immune-histochemistry staining (Castel and Morris, 1988; Yao et al., 2017). Some studies showed that the CPu has no fibers after injecting a virus reporter at both the PVN and SON in male mice. Here we found that some pass-through fibers are present in the CPu, which might go laterally to the En. Since the virus reporter injection at the PVN and SON did not reveal fibers in the CPu, it is possible that these fibers originated from other oxytocin-expressing neurons in the POA s or that a few oxytocin neurons in the PVN or SON, which are not easily labeled by virus reporters, do specifically target the rostral brain areas.

In the AcB (or NAc), we observed that there are far more oxytocin terminals in the AcbSh compared to the AcbC. However, since we did not have specific landmarks to label the boundary of AcbSh and AcbC, it is possible that most terminals are targeting the AcbSh. It has been shown that the AcB receives input mostly from PVN magnocellular cells and some SON oxytocin neurons in mouse (Yao et al., 2017). Unfortunately, here we could not verify the origin of the terminal branches in the AcB due to heavy staining near the SON and PVN regions. AcB is important for the reward system such as social rewards (Dölen et al., 2013; Dölen and Malenka, 2014). In prairie voles, the OXTR in AcB plays an important role in its monogamous and pair bonding. Although rats and mice are non-monogamous animals, the oxytocin processing and its connection to the AcB may still play a critical role in regulating social behavior. Furthermore, some branching and neurites might have connections with the mPFC area, which controls emotional social behavior (Ko, 2017).

### Innervation in Cortical and Olfactory Areas

It is well known that oxytocin is involved in many social behaviors in rodents such as social memory and sexual preference, which relies on the olfactory system. Here we confirmed that many oxytocin-Cre-labeled fibers are in the Pir cortex, AON, and TT regions. The input of oxytocin-expressing neurons in the olfactory areas and mPFC may together create some complicated mechanism to regulate social behavior and the reward system (Ferguson et al., 2001). Interestingly, we observed that terminal fibers in the Pir and insular cortex have some enlarged varicose-like structures with zigzag neurite patterns but branch much less compared to other regions. Therefore, additional investigation is required to verify the connection of oxytocin neuron in the insular and Pir. Finally, here we also observed from multiple subjects with sparse but clear branching terminals in the Cg area, which confirms previous report showing oxytocin fiber in Cg from lactating female rat (Knobloch et al., 2012). We found that oxytocin fiber density in the cortical and olfactory area together shows high individual variation.

### Innervation in Thalamus

Previous reports show inconclusive results of oxytocin fibers in the PV region of the thalamus. Some studies showed dense innervation, while some showed sparse innervation. Here we observed very sparse and thin branches in the PV. Moreover, the strength of the staining signal in the PV reveals high variation between different individuals, supporting previous reports. Since PV has been shown to regulate attention, movement, cognition, and emotions, it is likely that oxytocin modulation to these functions will have high individual variance.

### Innervation in Midbrain

It has been shown that oxytocin can modulate the social reward system or aversive behavior in VTA (Johns et al., 2011; Hung et al., 2017). Here we observed that most oxytocin-Cre-labeled bundles extend to the spinal cord through the midbrain. In addition, some branch-like terminals are in the VTA and PBP, suggesting direct innervation from the hypothalamus to the VTA. There are no branch-like terminals in the SN region. However, we observed some branches at the posterior part of the SNc sub-region, which is similar to the previous report (Xiao et al., 2017). In addition, we also observed some obviously branch-like terminals in the inferior colliculus, which supports the previous hypothesis that oxytocin might participate in modulating the sensory processing in *Pteronotus parnellii* (Kanwal and Rao, 2002). Finally, some fibers extend to the VLPAG and form branch-like terminals. There is some individual variation of oxytocin fiber density in the VTA and VLPAG regions.

### Innervation in Hindbrain and Medulla

Previous literature mentioned that some parvocellular oxytocin neurons project toward the medulla and spinal cord through the midbrain in rodents and humans (Sofroniew, 1980, 1983b; Sawchenko and Swanson, 1982; Jenkins et al., 1984). A previous study showed that oxytocin neurons in the PVN project to the LC in rats (Sofroniew, 1980), and local administration of oxytocin in the LC might modulate pain and anxiety processes. Here we found some thin oxytocin terminals in the LC and surrounding regions such as the Bar and PB areas. It was reported that lesion of the LC would impact oxytocin release induced by hemorrhage (Rodovalho et al., 2006).

Finally, here we report consistent oxytocin-Cre-labeled cell bodies and fibers in most brain regions in the hypothalamus, while some brain regions such as the cortical area, mPFC, PV, VTA, and PAG show high individual variation. Therefore, the oxytocin system may modulate many physiological functions through conserved basal circuitry and high plasticity circuits. Whether these two circuits are parallel or overlap with each other requires further investigation.

## Data Availability Statement

All datasets presented in this study are included in the article/Supplementary Material.

## Ethics Statement

The animal study was reviewed and approved by NTU IACUC.

## Author Contributions

P-YL and S-KC initiated the project and wrote the manuscript. P-YL, Y-MC, and J-HY performed the experiment and analyzed the result.

## Conflict of Interest

The authors declare that the research was conducted in the absence of any commercial or financial relationships that could be construed as a potential conflict of interest.

## Supplementary Material

Supplementary Table 1 Abbreviation list.

Supplementary Movie 1 Z stack movie of Figure 3F.

Supplementary Movie 2 Z stack movie of Figure 4B.

Supplementary Movie 3 Z stack movie of Figure 3E.

Supplementary Movie 4 Z stack movie of Figure 6C.
