# Supplementary material for: Mapping Central Projection of Oxytocin Neurons in Unmated Mice Using Cre and Alkaline Phosphatase Reporter
Source: Front Neuroanat. 2020 Oct 19;14:559402. doi: 10.3389/fnana.2020.559402 (PMC7604466; doi:10.3389/fnana.2020.559402)
Supplement: Supplementary Table 1 — Abbreviation list. [file Table_1.docx]

| Supplementary Table 1 Abbreviation list. | | | |
| --- | --- | --- | --- |
| 3V | 3rd ventricle | **MePV** | medial amygdaloid nucleus posteroventral part |
| 4V | 4th ventricle | **MHb** | medial habenular nucleus |
| AcbC | nucleus accumbens, core | **MiTg** | microcellular tegmental nucleus |
| AcbSh | nucleus accumbens, shell | **ML** | medial mammillary nucleus lateral part |
| aca | anterior commissure, anterior part | **MM** | medial mammillary nucleus medial part |
| AHA | anterior hypothalamic area, anterior part | **MnPO** | median preoptic nucleus |
| AHC | anterior hypothalamic area, central part | **MO** | medial orbital cortex |
| AHN | anterior hypothalamic nucleus | **MPB** | medial parabrachial nucleus |
| AHiAL | amygdalohippocampal area, anterolateral part | **MPOL** | medial preoptic nucleus lateral part |
| AHP | anterior hypothalamic area, posterior part | **MPOM** | medial preoptic nucleus medial part |
| AOM | anterior olfactory area medial part | **MS** | medial septal nucleus |
| Aq | aqueduct | **MVPO** | medioventral periolivary nucleus |
| Arc | arcuate hypothalamic nucleus | **och** | optic chaism |
| Bar | Barrington's nucleus | **opt** | optic tract |
| BIC | brachium of the colliculus | **PaAP** | paraventricular hypothalamic nucleus anterior parvocellular part |
| BLA | basolateral amygdaloid nucleus, anterior part | **PaMM** | paraventricular hypothalamic nucleus medial magnocellular part |
| BLP | basolateral amygdaloid nucleus, posterior part | **PaV** | paraventricular hypothalamic nucleus ventral part |
| BMA | basomedial amygdaloid nucleus, anterior part | **PBP** | parabrachial pigmented nucleus of the VTA |
| BST | bed nucleus of the stria terminalis | **Pe** | periventricular hypothalamic nucleus |
| CA1 | field CA1 hippocampus | **PH** | posterior hypothalamic nucleus |
| CAT | nucleus of the central acoustic tract | **PL** | paralemniscal nucleus |
| CEA | central amygdaloid nucleus | **Pir** | piriform cortex |
| CeC | central amygdaloid nucleus, capsular part | **PLCo** | posterolateral cortical amygdaoid area |
| CeL | central amygdaloid nucleus, lateral division | **PLH** | peduncular part of lateral hypothalamus |
| CeM | central amygdaloid nucleus, medial division | **PrL** | prelimbic cortex |
| Cg1 | cingulate cortex, area 1 | **PS** | parastrial nucleus |
| CIC | commissure of the inferior colliculus | **PV** | paraventricular nucleus of thalamus |
| Cnf | cuneiform nucleus | **PVN** | paraventricular nucleus of the hypothalamus |
| Cpu | caudate putamen (striatum) | **RChL** | retrochiasmatic nucleus, lateral part |
| D3V | dorsal 3rd ventricle | **RR** | retrorubral nucleus |
| DEn | dorsal endopiriform claustrum | **scp** | superior cerebellar peduncle |
| DLPAG | dorsolateral periaqueductal gray | **SCN** | suprachiasmatic nucleus |
| DM | dorsalmedial hypothalamic nucleus | **SHy** | septohypothalamic nucleus |
| DMPAG | dorsomedial periaqueductal gray | **SNC** | substantia nigra compact part |
| DP | dorsal peduncular cortex | **SNCD** | substantia nigra compact part dorsal tier |
| DPO | dorsal periolivary region | **SNL** | substantia nigra lateral part |
| DTT | dorsal tenia tecta | **SNR** | substantia nigra reticular part |
| ECIC | external cortex of the inferior colliculus | **SON** | supraoptic nucleus |
| Fmi | forceps minor of the corpus callosum | **SOR** | supraoptic nucleus |
| IC | inferior colliculus | **SPO** | superior paraolivary nucleus |
| IL | infralinbic cortex | **StHy** | striohypothalamic nucleus |
| InG | intermediate gray layer of the superior colliculus | **STLD** | bed nucleus of the stria terminalis lateral division, dorsal part |
| InWh | intermediate white layer of the superior colliculus | **STLP** | bed nucleus of the stria terminalis lateral division, posterior part |
| LC | locus coeruleus | **STLV** | bed nucleus of the stria terminalis lateral division, ventral part |
| LHA | lateral hypothalamic area | **STMPI** | bed nucleus of the stria terminalis medial division, posterointermediate part |
| LHb | lateral habenular nucleus | **STMPL** | bed nucleus of the stria terminalis medial division, posterolateral part |
| LM | lateral mammillary nucleus | **STMV** | bed nucleus of the stria terminalis medial division, posterolateral part |
| LO | lateral orbital cortex | **STMPM** | bed nucleus of the stria terminalis medial division, ventral part |
| LPAG | lateral periaqueductal gray | **SuVe** | superior vestibular nucleus |
| LPB | lateral parabrachial nucleus | **VCI** | ventral part of claustrum |
| LPO | lateral preoptic area | **VDB** | nucleus of the vertical limb of the diagonal band |
| LSI | lateral septal nucleus, intermediate part | **VLL** | ventral nucleus of the lateral lemniscus |
| LSO | lateral superior olive | **VLPO** | ventrolateral preoptic nucleus |
| LV | lateral ventricle | **VMH** | ventromedial hypothalamic nucleus |
| LVPO | lateroventral periolivary nucleus | **VMPO** | ventromedial preoptic nucleus |
| ME | median eminence | **VO** | ventral orbital cortex |
| MeA | medial amygdaloid nucleus anterior part | **VTA** | ventral tegmental area |
| MeAV | medial amygdaloid nucleus anteroventral part | **VTAR** | ventral tegmental area rostral part |
| MePD | medial amygdaloid nucleus posteroventral part | **VTM** | ventral tuberomammillary nucleus |
